# The Psychometric Properties of the Older People's Quality of Life Questionnaire, Compared with the CASP-19 and the WHOQOL-OLD

**DOI:** 10.1155/2009/298950

**Published:** 2010-02-01

**Authors:** Ann Bowling

**Affiliations:** Department of Primary Care and Population Sciences, University College London, Hampstead Campus, London NW3 2PF, UK

## Abstract

*Purpose*. To present the psychometric properties of a new measure of quality of life in older age, the Older People's Quality of Life (OPQOL) Questionnaire, compared with the CAPSE-19 and the WHOQOL-OLD. *Design and Methods*. The vehicle was three national population surveys of older people living at home in Britain, including a survey of ethnically diverse older people. *Results*. The OPQOL had acceptable levels of reliability and validity in British population samples of older people, but more modest in the ethnically diverse population sample. The CASP-19 and WHOQOL-OLD had acceptable levels of reliability and validity in the British population sample, but not in the ethnically diverse sample. *Implications*. The OPQOL has potential for use as a multidimensional population surveillance instrument for use with older populations, or as an outcome measure of multisector policy. Its strengths are that its development was embedded firmly in the perspectives of older people, integrated with theory.

## 1. Introduction

Increasing concerns about the policy implications of ageing populations, and increased life expectancies, in the developed world, have led to interest in interventions to improve older people's health, independence, activity, social and economic participation, thereby their *active* contribution to society—and also, in effect, adding *quality* to extended years. Assessment of the effectiveness of public policy in these areas requires the use of relevant and valid outcome measures. Assessment of quality of life (QoL) is a commonly used end-point of health technology assessment and is the stated end-point of policies aiming to promote active ageing [1].

However, the various models of quality of life are not consistent. Some have incorporated a needs-based satisfaction model, based on Maslow [2] and Maslow [3] hierarchy of human needs for maintenance and existence (physiological, safety and security, social and belonging, ego, status and self-esteem, and self-actualisation). Higgs et al. [4] and Hyde et al. [5] based their model of QoL in older age on self-actualisation and self-esteem. In contrast, traditional U.S. social science models of quality of life have been based primarily on the overlapping, positive, concepts of “the good life,” “life satisfaction,” “social well-being,” “morale” “the social temperature,” or “happiness” [6–8]. The focus among psychologists is on psychological resources [9]. Lack of agreement on a concept of QoL across disciplines has hindered attempts at multidimensional measurement.

Research with older populations has also suffered from the lack of generic QoL instruments that are truly applicable to this group. Investigators of QoL outcomes in older age have commonly applied the Short-From-36 health status questionnaire as a proxy for QoL, although some research suggests that older age groups have higher rates of item nonresponse, than others, with this instrument because they find several items not applicable to them [10, 11]. The multifaceted nature of QoL has posed particular challenges for measurement. Few measures of QoL are truly multidimensional, while ageing and increased frailty can have effects on several areas of life. As QoL is a largely subjective concept, it is essential to reflect lay views in any instrument designed to measure it. Most existing measures of QoL are based on theoretical concepts such as human need, life satisfaction, broader health, or are individualised and expensive to administer [12]. 

Recent attempts to address this gap in available measurement instruments for use with older people include the development of the CASP-19 and the WHOQOL-OLD. The CASP-19 (19 items) was based on models of needs satisfaction and self-actualisation, and aimed to measure Control, Autonomy, Self-realisation and Pleasure, although there was relatively little lay input into its construction [4, 5]. The WHOQOL-OLD (24 items) is a module of the World Health Organization's (WHO) broader measure of QoL, the WHOQOL, which was designed for adults of all ages. The WHOQOL-OLD includes additional items, judged by focus groups to be missing from the WHOQOL if applied to older people [13]. Despite the collection of a large amount of qualitative data, the WHOQOL group made a prior, largely “expert led” decision about the domains for inclusion in the WHOQOL; there was also little indication that the group had drawn on the broader QoL literature in the construction of the measure. 

The research presented in this paper intended to shift the paradigm of questionnaire development towards a more constructivist approach embedded firmly in the perspective of the older person, integrated with theory, embracing the epistemological challenge that lay views pose for academic theories. Social investigations can benefit from grounding in lay views, as they provide understandings in terms of cause and meanings, and they are a vehicle for people to reassert their worth (empowerment) [14]. By prior testing lay views against theoretical models, we also satisfied the condition for the development of measures that they are embedded in theory [15].

The QoL Survey, funded by ESRC Growing Older programme, was the first representative study of the QoL of people aged 65+, living at home in Britain. We asked 999 randomly sampled people aged 65+ open-ended interview questions about what gave their lives quality, what took quality away, and their priorities, followed by a self-rated QoL uniscale and a series of structured measures. We also followed-up 80 respondents and asked them about their QoL in-depth. The study was unique in obtaining quantitative *and *qualitative data on social, psychological, environmental, health, and personal circumstances from the *same* nationally representative sample of people aged 65+ in Britain. The unique, rich dataset led to a lay based, *multidimensional * model of social, economic, psychological, health and neighbourhood influences (both positive and negative) on QoL, and which overlapped with theoretical models taken in combination [16–19].

We adopted a *multidisciplinary* perspective and assessed theoretical influences on QoL using validated, structured measures of social, psychological morbidity, health and functioning, psychological resources, including self-efficacy and control; perceived neighbourhood social capital and facilities in the built environment, including transport, socioeconomic and social circumstances. In our survey, in order to separate these predictor from the component variables of QoL, QoL was considered as unidimensional construct (measured with a global QoL uniscale), but with *multiple influences*. Respondents emphasised the importance of social and psychological resources, health and functioning, neighbourhood resources, adequate finances and independence *for* a good QoL. These were categorised as main themes. The meanings underlying these were also coded (as subthemes). They cut across the main themes, emphasising the freedom to do the things they wanted to do without restriction: pleasure, enjoyment, and satisfaction with life; mental harmony; social attachment—having access to companionship, intimacy, love, social contact, and involvement, help; social roles; feeling secure. Respondents' statements which reflected the most commonly occurring themes and subthemes were included in an initial 50-item version of the Older People's Quality of Life Questionnaire (OPQOL). The full version of OPQOL was piloted for face validity and acceptability in focus group interviews and with over 100 volunteers from the original QoL survey respondents. Initial tests of internal consistency (Cronbach's alpha) and construct validity were also conducted at this stage, and which led to item-reduction. This process is described in more detail later under *Measures.*

The objective of the paper presented here is to describe the full psychometric testing, and psychometric properties, of the Older People's Quality of Life Questionnaire (OPQOL), and compare it with the CASP-19 and WHOQOL-OLD in samples of older people in the British population and an ethnically diverse sample.

## 2. Aim

To present the psychometric properties of the OPLQOL compared with the CAPSE-19 and the WHOQOL-OLD.

## 3. Methods

The study was based on three national surveys of older people living at home in Britain.

- People aged 65+ responding to two waves of the Ethnibus Surveys (http://www.ethnibus.com/) in 2008. This is a rolling face-to-face quota sample interview survey with adults aged 16+, living at home, based on a statistically robust sample of ethnic minority populations in Britain; the response rate was 70%.
- People aged 65+ responding to two waves of the Office for National Statistics (ONS) Omnibus Survey (http://www.statistics.gov.uk/) in 2008. This is a rolling face-to-face interview survey with adults aged 16+, living at home, based on a stratified random sample of postcodes across Britain; the response rate was 61%.
- A further postal followup in 2007-2008 of people living at home in Britain, aged 65+ in 1999-2000, who first responded to four waves of an ONS Omnibus, face-to-face interview survey, based on a stratified random sample of postcodes across Britain during 1999/2000; the follow-up response rate was 58%.

For more details of methods of sampling and response see Appendix 1 of methods in Supplementary Material available online at doi:10.1155/2009/298950.

### 3.1. Measures

The measures of QoL used included the newly developed Older People's Quality of Life Questionnaire (OPQOL), which was administered in all three surveys; and, in the two face-to-face interview surveys only, the CASP-19 [4, 5] and the WHOQOL-OLD (derived from the WHOQOL) [13]—as this mode is the least cognitively taxing for respondents, permitting a longer questionnaire [12]. In addition, standard sociodemographic items were included, and questions on active ageing, QoL, health, and psychosocial circumstances were asked in the baseline QoL survey [12].

The CASP-19 (Control, Autonomy, Self-realisation and Pleasure) was developed from the theory of human needs satisfaction, and tested with focus groups and a survey of people aged 65–75 [4, 5]. It concentrates on four theoretically derived (19 items): Control (4 items), Autonomy (5 items), Pleasure (5 items), Self-realisation (5 items), with four-point Likert response scales “Often” to “Never.” Items are scored (with reverse coding of positive responses, so that higher scores equal higher QoL; the authors define the scale ranges as 0 (complete absence of QoL) to 57 (total satisfaction in all four domains).

The WHOQOL-OLD was developed from the parent instrument: the World Health Organization's WHOQoL Group's WHOQOL-100, and cross-cultural studies; it was tested on convenience samples of older people across cultures [13]. It is a multidimensional measure of QoL and comprises seven subscales (24 items): sensory abilities, autonomy, past present and future activities, social participation, death and dying, and intimacy (4 items per subscale). Items are scored with reverse coding of positive responses, so that higher scores equal higher QoL; the authors define the scale ranges as 24 (lowest possible QoL) to 120 (highest possible QoL). Response scales are all 5-point but vary in their wording (“Not at all” to “An extreme amount”/“Completely”/“Extremely;” “Very poor” to “Very good;” “Very dissatisfied” to “Very satisfied;” “Very unhappy to Very happy”).

The OPQOL is a new 32- to 35-item QoL measure. It has 5-point Likert scales from Strongly Agree to Strongly Disagree, wih 32 or 35 items, representing: life overall (4 items), health (4 items), social relationships and participation (7 items in QoL follow-up survey, 8 items in Omnibus surveys), independence, control over life, freedom (5 items), area: home and neighbourhood (4 items), psychological and emotional well-being (4 items), financial circumstances (4 items), and religion/culture (2 items; asked in Omnibus surveys only). Items are scored (with reverse coding of positive responses, so that higher scores equal higher QoL; the scale ranges are 35 (QoL so bad could not be worse) to 175 (QoL so good could not be better) (Omnibus surveys) and correspondingly 32 to 160 in the QoL follow-up survey. 

As stated earlier, the OPQOL was conceptually grounded in lay views from the baseline QoL Survey, integrated with theory from a synthesis of the literature. First, older people's responses to open-ended questioning about the “good things” that gave life quality were examined. These were categorised into main themes by two researchers, independently. These were, in order of magnitude: social relationships (mentioned by 81%), social roles and activities (60%), solo activities (48%), health (44%), psychological outlook and well-being (38%), home and neighbourhood (37%), financial circumstances (33%), and independence (27%). Smaller numbers mentioned various other things. These responses were consistent with older people's views about what took quality away from life. Poor health was most often mentioned as the thing that took “quality away” from their lives (by 50%). Other commonly mentioned things that took quality away from life were home and neighbourhood (30%), financial circumstances (23%), and psychological outlook (17%). Having health, followed by better finances (i.e., having enough/more money), were the two most frequently mentioned things that respondents said would improve the quality of their own lives. The subscale domains in the OPQOL reflected this common core of main constituents of quality of life. The pool of *actual verbatim responses* was examined next by two researchers, again independently, to inform the inclusion of the items within each subscale. The main *reasons *given by people, at survey and in-depth interview, to explain the importance of these themes to their QoL were categorized, by two independent coders, as freedom to do the things they wanted to do without restriction (whether in the home or socially); pleasure, enjoyment, and satisfaction with life; mental harmony; social attachment—having access to companionship, intimacy, love, social contact and involvement, help; social roles; and feeling secure. These cut across the main themes [17]. The responses which were selected for inclusion in OPQOL represented the most commonly occurring subthemes within each theme. 

The verbatim responses formed an initial pool of over 100 different statements, or attitudes. After reading and comparing the items, overlapping statements were deleted to leave 51 items. The revised items were first mailed to QoL Survey sample members in 2006 and 60% 179 of the respondents invited to participate returned the completed questionnaires. They were asked to complete the items, report any difficulties they had with it, and to make any other comments about it. Psychometric tests for item redundancy, reliability, and validity led to the removal of redundant items (over-high correlations), items with high missing data, items where the Cronbach's alpha of the scale improved with their removal, items which did not correlate with the overall scale score or a self-rated global QoL item. Amendments to wording were made following feedback from survey respondents and an opportunistic focus group of eight consenting people aged 65+ whose role locally was to provide feedback on research and services (seven of whom were white). This resulted in a reduced 32-item, multi-dimensional QoL questionnaire, with the methodological advantage that it separates constituents of QoL from QoL end states.

The questionnaire was further assessed for interpretation, face, and content validity with four focus groups of older people, three of which reflected ethnic diversity, and were organised by Ethnibus's focus group arm before the Ethnibus and ONS Omnibus waves commenced (http://www.ethnifocus.com/). Participants were asked about their understanding of the concepts underlying the term quality of life, were told there were no right or wrong answers, and instructed to mention as many things as they wished. They were also asked to comment on the meanings of the questionnaire items.

In 2007-2008, the QoL follow-up survey sample was administered the postal questionnaire, containing the pretested 32-item version of the OPQOL, with 5-point Likert scales from “Strongly Agree” to “Strongly Disagree.” The ONS Omnibus and Ethnibus surveys were face-to-face home interviews and respondents were administered a slightly longer 35-item version, which included three additional items derived from the views of members of the three later Ethnifocus focus groups. These items were added to ensure greater relevance of the measure to ethnically diverse sample members (the Ethnibus focus groups unfortunately took place after the QoL follow-up postal survey had been administered).

### 3.2. Statistical Analysis

Univariate analyses included chi-square tests, and psychometric tests for validity and reliability. For homogeneity, or internal consistency, the tests used were Cronbach's alpha test of the strength of the association between each scale item and the full scale, item-item and item-total reliability correlations (using SPSS _13_ reliability function), intraclass correlations for test-retest reliability, and validity (using Spearman's rank correlations with theoretically relevant variables). Multiple regression analysis was used to examine the independent predictive ability of theoretically relevant variables on the QoL measures, which had achieved statistical significance with univariate analyses at least at the 0.05 level. A hierarchical approach was used, with independent variables entered manually in their theoretical order of importance. This is a preferred method over data-driven techniques of variable selection [20, Page 115]. The level for statistical significance was set at *P* < .05. The level for statistical significance was set at *P* < .05. The variables entered did not correlate by more than 0.732, and tests for multicollinearity were satisfied. Standard socioeconomic variables were also entered on the basis of their a priori significance, to control for their effects. Tests of internal consistency, including Cronbach's alpha (criteria of acceptability 0.70 < 0.90), were applied to the data in order to assess the strength of the association between each scale item and the full scale. *The test-retest reliability of the OPQOL was assessed by mailing a second copy of the questionnaire, plus items about any recent life changes, to a subsample of 50 consenting QoL Longitudinal Survey respondents four weeks after they had returned the first questionnaire). *Construct (*convergent and discriminant*) validity was tested by assessing the strength of Spearman's rho correlations between the scales and similar or relevant/dissimilar measures (the QoL domain ratings and additional questionnaire items). Factor analysis was used to examine the dimensions underlying the questionnaire, including the criteria that the correlation matrix should reveal many coefficients of 0.30 and above; the Kaiser-Meyer-Olkin Measure of Sampling Adequacy (KMO) [21, 22] should exceed the threshold of >0.60; the Bartlett's (1954) Test of Sphericity should be statistically significant at *P* = .001 in order to support the factorability of the correlation matrix, and suggest that the use of factor analysis is appropriate. Eigenvalues should exceed the threshold of >1.0 to support the construct validity of the scale.

## 4. Results

### 4.1. Characteristics of Samples

Over half of each sample (52–54%) comprised women. While 91% (363) of the Ethnibus sample were aged 65 < 75 (in reflection of the younger age distributions of ethnic populations in Britain), 55% (326) of the ONS Omnibus sample, and 17% of the QoL follow-up survey, were aged 65 < 75. The remainder were all aged 75+. In reflection of their younger age, more of the Ethnibus sample were married/cohabiting than widowed (58%, 230) compared with 49% (285) and 49% (138) of the ONS Omnibus and QoL follow-up samples, respectively. Fewer Ethnibus respondents were owner occupiers, (52%, 208) than other sample members, 73% (429) and 85% (239), respectively, and they were more likely to live with friends or family. They had the largest numbers of adults per household (30%, 118) lived in households with more than four people aged 18+, compared with 1% (%) and none of the ONS Omnibus and QoL follow-up respondents). In contrast just 5% (19) of Ethnibus respondents lived alone, while about half of the ONS and QoL follow-up samples, 48% (286) and 49% (137), respectively, lived alone. Few of the ONS Omnibus survey members were members of ethnic minority groups, reflecting the distributions in the national population, as did none of the QoL follow-up sample members. These differences should be noted when comparing sample distributions.

### 4.2. Scale Acceptability

An open-ended postal questionnaire item asked respondents how easy or difficult they found the OPQOL to complete, and comments indicated they found it very easy and acceptable. Item-completion was at acceptable levels—item nonresponse for all three QoL scales was between 1 < 3% in both interview surveys, although, as expected, slightly higher in the self-administered postal survey with the older QoL follow-up sample (5–10%—one item about having paid or unpaid work/activities that give a role in life reached 11%). 

Table 1 summarises the total summed scale distributions, and means, for the samples. Members of the Ethnibus sample had consistently poorer QoL on the three QoL measures, followed by older QoL follow-up sample members, compared with the ONS Omnibus sample who had the best QoL. These differences are highly significant. The distributions of grouped scale scores on each instrument showed a tendency to span middle values, although, as expected, these were more distributed towards middle-poor QoL for the Ethnibus sample, and towards middle-good QoL for the British population sample. Few OPQOL scores were distributed at the extreme ends, although this reflected the wider score ranges within the OPQOL, compared with CASPE-19 and WHOQOL-OLD.

The detailed responses for each sample on the three QoL measures (OPQOL, CASP-19, and WHOQOL-OLD) are shown in Tables 1–3 in Supplementary Material. These distributions also show that responses to most items spanned the full range, although more Ethnibus respondents opted for middle categories, thus “sitting on the fence,” compared with other respondents, whose responses were more positive. For scale acceptability, floor and ceiling effects (responses at top and bottom ends of the measure) should ideally be <20%, although this standard is difficult to achieve in research on well-being, life satisfaction, and QoL, where some positivity bias is known to occur. Despite the use of 5-point response scales in the OPQOL and WHOQOL-OLD, and the use of 4-point response scales in the CASP-19, some responses exceeded this level. These tables also show that the OPQOL items were all consistently more likely to discriminate between the samples then the CASP-19 or WHOQOL-OLD items.

The test-retest reliability of the OPQOL was assessed by mailing a second copy of the questionnaire, plus items about any recent life changes, to a subsample of 50 consenting QoL Longitudinal Survey respondents four weeks after they had returned the first questionnaire; 31 respondents returned the questionnaire within the required time period. Four week, intraclass test-retest correlations for the OPQOL ranged between 0.403 and 0.782, with the lower correlations being explained by reported life changes in the intervening month, demonstrating the difficulties of test-retest exercises in older populations, and the need for shorter test-retest periods.

### 4.3. Reliability

Table 1, earlier, showed that the Cronbach's alpha statistic met the 0.70 < 0.90 (for internal consistency without item redundancy) threshold for each QoL measure for the OPQOL in each sample, but, in contrast to the OPQOOL, the CASP-19 and WHOQOL-OLD failed to meet this criteria for the Ethnibus sample, although item responses were consistent with the OPQOL responses in the Ethnibus sample. Cronbach's alpha statistic is sensitive to the magnitude of correlations among items and the number of items included in the scale [23], with the effect that the alpha is usually higher the greater the number of scale items. This usually affects small scales of <10 items. It is unlikely that this would account for the stronger alpha of the OPQOL (32 and 35 items), particularly with the Ethnibus sample, compared with the CASP-19 (19 items) or the WHOQOL-OLD (24 items). 

Subscales for the OPQOL and CASP-119 met criteria for correlations with the total scale (*r* > 0.20), except the religion/culture subscale in the ONS Omnibus sample, perhaps reflecting the lip-service generally paid to religion in the total British population. However, few of the WHOQOL-OLD subscales met the criteria (notably DAD subscale in both samples and all but the PPF subscale failed the criteria in the Ethnibus sample) (see Tables 4–6 in Supplementary material).

### 4.4. Validity

There is no gold standard QoL measure to assess criterion validity, but construct validity (convergent and discriminant) validity was tested. Consistent with the literature [12], Table 4 shows that the OPQOL and WHOQOL-OLD correlated with active ageing, health, and functioning, as would be expected. Each QoL measure, with the exception of the WHOQOL-OLD in the Ethnibus sample, was significantly correlated with self-rated active ageing, with respondents reporting optimum levels of active ageing having better (higher) QoL scores.

More optimal QoL scores were obtained by those with better health and functional ability. However, several of the CASP-19 items failed to correlate with health and functioning in the Ethnibus sample. The minus signs reflect the different directions of coding. Also, as expected, the availability of informal help and support was significantly associated with the OPQOL in each sample, with more available people correlating with higher QoL, although the associations were frequently not significant with the CASP-19 and WHOQOL. Older age was inversely associated with QoL on each measure across samples, with younger people having a better QoL. There were no associations with sex, as expected [12]. Socioeconomic variables were significantly correlated with the OPQOL, although less often with the CASP19 and WHOQOL-OLD. Marital status was also significantly correlated with the OPQOL in the Omnibus sample only, with married people having a better QoL than unmarried people (see Table 4). 

These results support the construct validity (convergent and discriminant) of the OPQOL in each sample, but only partly support that for the WHOQOL-OLD and CASP-19 which performed best in the ONS Omnibus survey rather than the ethnically diverse Ethnibus sample.

The construct validity of the three QoL measures was also tested by correlating them with independent self-rated QoL and self-ratings on several of its domains. Before the three QoL scales were administered, respondents were asked to rate their QoL overall, and in relation to their health, social relationships, independence, control and freedom, home and neighbourhood, psychological/emotional well-being, financial circumstances and leisure, and social activities. The correlations between their global and domain ratings and the OPQOL, CASP-19, and WHOQOL-OLD are shown in Table 2. The OPQOL was highly significantly associated with the global and domain ratings in each of the three samples in the expected directions (better self-ratings correlated with better QoL scores). The CASP-19 and WHOQOL-OLD correlated significantly with global QoL self-ratings in each sample, and with most of the domain self-ratings in the ONS Omnibus sample, but often failed to correlate significance with the domain self-ratings in the Ethnibus sample.

Scale-scale and subscale-subscale correlations were also conducted between the OPQOL, CASP-19, and WHOQOL-OLD to assess construct validity further. It was expected that the scales and subscales, where a comparable domain of QoL was assessed, would correlate significantly with each other. Higher correlations would not necessarily be expected as the content of each measure differed. Table 3 shows that the OPQOL, CASP-19, and WHOQOL-OLD total scores all correlated moderately to highly with each other (rho: 0.380–0.732; all *P* < .01).

Tables 7–9 in the Supplementary Material show the results for the subscale-subscale correlations. The OPQOL subscales correlated significantly with all the CASP-19 subscales, except with OPQOL religion/culture, in the ONS Omnibus sample; there were fewer significant correlations in the Ethnibus sample (see Table 7 in Supplementary Material). The OPQOL and WHOQOL-OLD subscales correlated significantly for the ONS Omnibus sample, with the exception of WHOQOL-OLD DAD, but were less likely to correlate significantly with the Ethnibus sample (see Table 8 in Supplementary Material). Significant scale to scale and subscale to subcorrelations, in expected directions, between the WHOQOL-OLD and the CASP-19 were achieved for all subscales in the ONS Omnibus survey, but, again, not all correlated significantly in the Ethnibus survey (see Table 9 in Supplementary Material). 

The final test of construct validity was subscale to subscale correlations (tested with Spearman's rho) within each of the three QoL measures. Subscales within each QoL measure correlated significantly, using Spearman's rho, when expected theoretically. 

As expected, the OPQOL Psychological well-being and outlook subscale correlated significantly with the OPQOL Life overall subscale in the Ethnibus, ONS Omnibus, and QoL follow-up samples (Spearman's rho: 0.232, 0.554, 0.380 in each sample, resp.; all *P* < .01). Similarly, given that poor health and frailty can limit one's independence, the OPQOL health and functioning subscale correlated significantly with the OPQOL Control, independence, and freedom subscale: Spearman's rho: 0.138, 0.489, 0.460 (all *P* < .01) in the three samples, respectively. With just one exception (control to self-realisation—rho: 0.079) the CASP-19 subscales all inter-correlated significantly (between rho 0.160 to 0.835, all *P* < .01). In the WHOQOL-OLD, Past, present, and future abilities subscale correlated in each sample with Social participation subscale: rho 0.209 (Ethnibus) and 0.584 (ONS Omnibus) (both *P* < .01). Also, the WHOQOL-OLD self-realisation subscale correlated with the Pleasure subscale: rho 0.189 and 0.523 in the two samples, respectively (both *P* < .01).

All subscale to total score Spearman's rho correlations, for each measure within each sample, were highly significant at *P* < .01. The subscales to total correlation ranges for the three QoL measures, across samples, were: OPQOL rho 0.235 to 0.786 (all *P* < .01); CASP-19 rho 0.549 to 0.834; WHOQOL-OLD rho 0.291–0.761.

### 4.5. Factor Analysis

The 35 items of the OPQOL in the ONS Omnibus Survey, which contained the largest number of cases, were subjected to principle components analysis, using SPSS, in order to examine factor structure. Nunnally (1978) recommended at least 10 cases per item to be factor analysed, although at least 5 cases per item have been judged to be acceptable by others [24]. The suitability of the data was initially assessed for their suitability for factor analysis. The correlation matrix revealed that many of the correlations were 0.3 or above. The Kaiser-Meyer-Oklin value of sampling adequacy was 0.893, exceeding the recommended value of 0.6 [21, 22]. Bartlett's Test of Spherity [25] was statistically significant (Chi-square 7169.875, 595 degrees of freedom, *P* < .001), supporting the factorability of the correlation matrix.

PCA revealed the presence of nine components where the eigenvalues exceeded 1, and which explained cumulatively 60.583% of the total variance in QoL between respondents; component 1 explained the largest proportion of the variance, 24.052, supported by inspection of the screeplot [26]. Using the Kaiser criterion of retaining all components with eigenvalues above 1, most items (*n* = 27/35) loaded strongly (0.4+) on the first component; eight items loaded strongly (0.4), or moderately (3.0+) on the second component; 11 items loaded strongly-moderately on the third component; two items each loaded strongly on components 4–9. While this reflected the multidimensional structure of the OPQOL, more detailed examination, and confirmatory factor analysis with rotation, of the OPQOL is required before its factor structure can be confirmed.

## 5. Discussion

There is no widely accepted standard measure of QoL for use with older populations. Investigators usually apply a battery of functional, psychological, and broader health status measures to this group. More recently the CASP-19 and WHOQOL-OLD have been developed. The former is a theoretically derived measure, while the latter was based on the WHOQOL-100, which was developed for all adults with the inclusion of additional items, after feedback from focus groups. The OPQOL is a measure of quality of life which covers the domains nominated by, and initially piloted with, a national British sample of people aged 65+. It is the first multidimensional measure of QoL which is derived directly from lay people's views of what gives their lives quality, and what takes quality away. The lay concepts were also compared with a wide range of theories of QoL, and judged to overlap and complement each other [16, 17].

Overall, the OPQOL met the thresholds for acceptability, internal consistency, and construct validity in British population samples of older people. In the ethnically diverse population sample, the results were more modest. The CASP-19, and WHOQOL-OLD also had acceptable levels of reliability and validity in the British population sample, but not in the ethnically diverse sample. Culture is likely to influence understandings of QoL to a large extent, and the weaker QoL correlations in the Ethnibus sample reflects this. More detailed examination of the structure of the OPQOL, including confirmatory factor analysis with rotation, is required before its factor structure can be confirmed.

The end columns of Tables 4–6 in the Supplementary Material show the corrected inter-subscale-total reliability correlations for the OPQOL, CASPE-19 and WHOQOL-OLD respectively. In both the British population sample aged 65+, and the older QoL follow-up sample, the OPQOL had strong inter-subscale-total correlations of over 0.60 for social relationships and participation, control, independence, freedom, and life overall. There was no directly comparable subscale for the CASPE-19 which focused its items and subscales on measuring control, autonomy, self-realisation, and pleasure. These achieved strong inter-subscale-total correlations in the British population sample of over 0.60 for the first three subscales (pleasure was just slightly lower at 0.547). The WHOQOL-OLD had one such correlation which exceeded 0.60 in the British population sample—present and future activities; the correlation for social participation in this sample was 0.551. However, the correlation for intimacy was lower at 0.310. While each of the three QoL instruments included indicators of control and related concepts, only the OPQOL included items detailing actual and desired social relationships, and of life overall. Given that the measure was developed directly from older people's understandings of QoL, the OPQOL may be a more sensitive indicator of the latter concepts. The lay relevance of the questionnaire also suggests that these concepts are important components of QoL, in contrast to the CASPE-19 which is based on a model of the primary importance of self-actualisation and self-esteem.

The weaknesses of the surveys needs to be considered when assessing the results. While response was good, there was still a substantial proportion of nonresponders to the ONS and QoL follow-up surveys, which is inevitably a cause for concern—especially with the longitudinal survey where the frailest drop out and/or die. However, ONS judged their ONS Omnibus respondents to be representative of people in Britain as a whole, when comparing them with population estimates based on the last census. In relation to the ONS interviews, interviewers made three calls, at different times of day, to each sampled household where no contact was made, before recording nonresponse. Response to national population surveys has been in decline over the past decade, and further research needs to investigate ways of enhancing response, including financial incentives for participation (this is not generally used in the UK).

Another weakness of the study design was the use of quota sampling, in targeted areas with ethnic minority populations, to achieve the Ethnibus sample. There are no ideal methods of sampling to reach these groups. The OPQOL also needs assessment of test-retest reliability in a larger sample, and with shorter time frames. 

In conclusion, with these cautions, the OPQOL has potential for use as a multidimensional population surveillance, or survey, instrument for use with older populations, or as an outcome measure of multisector policy, for example, aiming to promote well-being and more active ageing. It is unknown how the OPQOL would perform in specific patient or client groups, although its performance was good in national population samples aged 65+ and 74+, although more modest in the ethnically diverse population. The performance of the OPQOL was still stronger in the latter sample than the CASPE-19 or WHOQOL-OLD. This was possibly due to its assessment with ethnically diverse focus groups, and the consequent inclusion of two additional items on culture and religion which strengthened its performance in this sample (35-item version). Although further testing is required, the OPQOL is one of the small number of emerging measures to be considered when assessing the QoL of older people.

## Supplementary Material

Appendix 1 in the Supplementary Material show the methods of sampling and response.
Tables 1–3 in Supplementary Material show the detailed responses for each sample on the three QoL measures (OPQOL, CASP-19, and WHOQOL-OLD).
Tables 4–6 in Supplementary Material show the corrected inter-subscale-total reliability correlations for the OPQOL, CASPE-19 and WHOQOLOLD respectively.
Tables 7–9 in Supplementary Material show the results for the subscale-subscale correlations.

## Figures and Tables

**Table 1:** OPQOL, CASP-19, and WHOQOL-OLD distributions. NB. 3 additional OPQOL items were generated by Ethnibus focus groups which were too late to be included in the QoL follow-up survey, but were included in the ONS Omnibus and Ethnibus Surveys.

	Ethnibus survey (ethnically diverse British pop. aged 65+)	ONS Omnibus survey (British pop. aged 65+)	QoL follow-up survey (ONS Omnibus sample of British pop. aged 65+ in 1999/2000 refollowed up when aged 74+)
OPQOL TOTAL [ethnibus, ONS Omnibus 35 items; 5-point scale (1–5) range 35–175; QoL followup: 32 items, range 32–160)]	% (n)	% (n)	% (n)
≤ 99 QoL bad as can be	6 (24)	1 (6)	7 (17) ****
100–119	67 (266)	11 (64)	38 (96)
120–139	25 (100)	52 (289)	43 (108)
140–159	2 (9)	32 (178)	12 (29)
160–175 QoL good as can be	— (1)	4 (23)	—
*Mean (standard deviation)*	*114.538 (10.718)*	*134.730 (14.243)*	*121.385 (14.048)*
*Cronbach's alpha of internal consistency (reliability)*	*0.748*	*0.876*	*0.901*
No. responses	400	560	250
Casp-19 TOTAL [19 items; 5-point (0–3) scale range 0–57]			
≤19 absence of QoL	— (2)	1 (5) ****	n/a
20–29	23 (92)	7 (38)	
30–39	68 (271)	27 (158)	
40–49	8 (32)	46 (265)	
50–57 satisfaction in all domains	1 (3)	19 (107)	
*Mean (standard deviation)*	*33.235 (5.103)*	*41.836 (8.120)*	
*Cronbach's alpha of internal consistency (reliability)*	*0.553*	*0.866*	*n/a*
No. responses	400	573	
WHOQOL-OLD TOTAL [24 items; 5-point (1–5) scale range 24–120]			
≤69 lowest possible QoL	2 (6)	4 (22)****	n/a
70–79	23 (94)	11 (58)	
80–89	58 (234)	24 (130)	
90–99	15 (59)	40 (182)	
100–120 the highest possible QoL	2 (7)	27 (144)	
*Mean (standard deviation)*	*83.488 (6.547)*	*91.925 (11.764)*	
*Cronbach's alpha of internal consistency (reliability)*	*0.415*	*0.849*	*n/a*
N. responses	400	536	

*n/a: not asked in QoL follow-up survey as additional scales were judged to be too burdensome in the postal administration survey.*

****P < .0001.

**Table 2:** Construct validity of the OPQOL (32–35) CASP-19 WHOQOL-OLD (24): correlations with sociodemographic characteristics and circumstances (Spearman's rho).

	OPQOL TOTAL++	CASP-19 TOTAL++	WHOQOL-OLD TOTAL++
Self-assessed active ageing			
Ethnibus	−0.358**	−0.241**	−0.069
ONS Omnibus	−0.504**	−0.469**	−0.439**
QoL followup	−0.575**	n/a	n/a
Self-rated health status (5-point response scale Excellent-Poor)			
Ethnibus	−0.364**	−0.238**	−0.138**
ONS Omnibus	−0.543**	−0.530**	−0.465**
QoL followup	−0.628**	n/a	n/a
Level of ability to walk 400 yards *(4-point response scale No difficulty to unable to do alone) *			
Ethnibus	−0.207**	−0.095	−0.153**
ONS Omnibus	−0.446**	−0.488**	−0.331**
QoL followup	−0.507**	n/a	n/a
Level of ability to do heavy housework *(4-point response scale No difficulty to unable to do alone) *			
Ethnibus	−0.131**	−0.113*	−0.126*
ONS Omnibus	−0.456**	−0.520**	−0.364**
QoL followup	−0.465**	n/a	n/a
Level of ability to go shopping and carry heavy bags *(4-point response scale No difficulty to unable to do alone) *			
Ethnibus	−0.103**	−0.092	−0.108*
ONS Omnibus	−0.411**	−0.472**	−0.350**
QoL followup	−0.462**	n/a	n/a
Level of ability to go up and down stairs *(4-point response scale No difficulty to unable to do alone) *			
Ethnibus	−0.198*	−0.086	−0.128*
ONS Omnibus	−0.388**	−0.468**	−0.321**
QoL followup	−0.466**	n/a	n/a
No. of relatives who would help if needed with everyday chores, running errands, odd jobs:			
Ethnibus	−0.008	−0.058	−0.033
ONS Omnibus	0.163**	0.112**	0.177**
QoL followup	0.219**	n/a	n/a
No. of friends who would help if needed with everyday chores, running errands, odd jobs:			
Ethnibus	0.204**	0.089	0.102*
ONS Omnibus	0.403**	0.383**	0.381**
QoL followup	0.325**	n/a	n/a
No. of neighbours who would help if needed with everyday chores, running errands, odd jobs:			
Ethnibus	0.066	−0.011	0.017
ONS Omnibus	0.332**	0.308**	0.304**
QoL followup	0.171*	n/a	n/a
In a serious personal crisis, number of people could turn to for comfort and support:			
Ethnibus	0.112	−0.002	0.017
ONS Omnibus	0.270**	0.251**	0.300**
QoL followup	0.372**	n/a	n/a
Age of respondent (continuous)			
Ethnibus	−0.078	−0.062	−0.035
ONS Omnibus	−0.138**	−0.193**	−0.184**
QoL followup	−0.252**	n/a	n/a
Sex of respondent (Male vs. Female)			
Ethnibus	−0.028	−0.019	0.024
ONS Omnibus	−0.034	−0.032	−0.041
QoL followup	0.104	n/a	n/a
Socioeconomic Classification ranked++			
Ethnibus	−0.006	0.112*	−0.005
ONS Omnibus	−0.134*	−0.093*	−0.136**
QoL followup	−0.193**	n/a	n/a
Housing tenure (owner occupier/mortgage vs. rented)			
Ethnibus	−0.130**	−0.135**	−0.093
ONS Omnibus	−0.194**	−0.142**	−0.188**
QoL followup	−0.061	n/a	n/a
Has access to car/van in household (Yes vs. No)			
Ethnibus	n/a	n/a	n/a
ONS Omnibus	−0.189**	−0.176**	−0.197**
QoL followup	−0.199**	n/a	n/a
Marital status (married vs. unmarried)			
Ethnibus	−0.015	−0.032	0.015
ONS Omnibus	−0.116**	−0.084*	−0.153**
QoL followup	−0.182**	n/a	n/a

*++ ONS Omnibus and QoL followup: NS-SEC; Ethnibus Market Research Society classification*

*n/a: not asked*

*P < .05

**P < .01

**Table 3:** Validity OPQOL (35) CASP-19 WHOQOL-OLD (24) scores: correlations with QoL domain ratings (Spearman's rho).

	OPQOL TOTAL++	CASP-19 TOTAL++	WHOQOL-OLD TOTAL++
QoL domain ratings+:			
QoL as a whole			
Ethnibus (*n* = 400)	−0.389**	−0.273**	−0.128**
ONS Omnibus (*n* = 558–560)	−0.602**	−0.577**	−0.466**
QoL follow-up (*n* = 288)	−0.659**	n/a	n/a
			
Health			
Ethnibus	−0.148**	−0.135**	−0.042
ONS Omnibus	−0.624**	−0.576**	−0.450**
QoL followup	−0.628**	n/a	n/a
Social relationships			
Ethnibus	−0.159**	0.007	0.024
ONS Omnibus	−0.570**	−0.517**	−0.466**
QoL followup	−0.605**	n/a	n/a
Independence, control and freedom			
Ethnibus	−0.179**	−0.113*	−0.042
ONS Omnibus	−0.612**	−0.563**	−0.497**
QoL followup	−0.631**	n/a	n/a
Home and neighbourhood			
Ethnibus	−0.401**	−0.072	−0.075
ONS Omnibus	−0.526**	−0.426**	−0.418**
QoL followup	−0.439**	n/a	n/a
Pyschological/emotional well-being			
Ethnibus	−0.396**	−0.178**	0.004
ONS Omnibus	−0.567**	−0.510**	−0.484**
QoL followup	−0.675**	n/a	n/a
Financial circumstances			
Ethnibus	−0.243**	−0.093	−0.045
ONS Omnibus	−0.524**	−0.417**	−0.360**
QoL followup	−0.547**	n/a	n/a
Leisure and social activities			
Ethnibus	−0.338**	−0.115*	−0.094
ONS Omnibus	−0.639**	−0.593**	−0.512**
QoL followup	−0.606**	n/a	n/a

*P < .05; **P < .01

*+ Domain ratings: 5-point response scales Very good (1)–Very bad (5), with low scores representing optimum QoL ratings.*

*++ OPQOL, CASP-19, WHOQOL-OLD negative scores reversed so high scores represent optimum QoL.*

**Table 4:** OPQOL CASP-19 WHOQOL-OLD total scale scores correlation matrix (Spearman's rho): validity.

	OPQOL [35 items]	CASP-19 [19 items]	WHOQOL-OLD [24 items]
OPQOL			
Ethnibus	—	0.488**	0.405**
ONS Omnibus		0.732**	0.698**
CASP-19			
Ethnibus	0.488**	—	0.380**
ONS Omnibus	0.732**		0.694**
WHOQOL-OLD			
Ethnibus	0.405**	0.380**	—
ONS Omnibus	0.698**	0.694**	

**P < .01.
