# Supplementary material for: The Psychometric Properties of the Older People's Quality of Life Questionnaire, Compared with the CASP-19 and the WHOQOL-OLD
Source: Curr Gerontol Geriatr Res. 2010 Feb 1;2009:298950. doi: 10.1155/2009/298950 (PMC2819744; doi:10.1155/2009/298950)
Supplement: Supplementary file 1 — Appendix 1 in the Supplementary Material show the methods of sampling and response. Tables 1–3 in Supplementary Material show the detailed responses for each sample on the three QoL measures (OPQOL, CASP-19, and WHOQOL-OLD). Tables 4–6 in Supplementary Material show the corrected inter-subscale-total reliability correlations for the OPQOL, CASPE-19 and WHOQOLOLD respectively. Tables 7–9 in Supplementary Material show the results for the subscale-subscale correlations. [file 298950.f1.pdf]

Supplementary files of methods

www Supplementary file Appendix 1 of methods

Response rates by survey

Ethnibus Survey

Ethnibus is a monthly nation-wide survey of the main ethnic minority communities living in the UK (Indian, Pakistani, Bangladeshi, Caribbean, African and Chinese). Interviews were conducted by trained, multi-lingual field-workers. The Ethnibus Omnibus Survey was based on focused enumeration and stratification random sampling to ensure that samples are representative of the population. For sampling, Ethnibus used Census information on ethnicity across postal sectors, and listed the postal sectors according to concentration. Systematic random sampling was then used to ensure an even spread of postal sectors with differing concentrations. The number of addresses selected within the sector were proportional to the size of the ethnic concentration, e.g. high concentration sector would yield high number of interviews. These addresses form the starting point of the focused enumeration procedure.

Ethnibus targeted the following, most common, ethnic groups: Indian Pakistani, Caribbean, Chinese people. Sample boosting on the doorstep was used to include greater numbers of people aged 65 and over. Interviews were obtained until target was achieved.

A total of 400 interviews were aimed for and were obtained (200 per wave agreed, two waves required to achieve 400). The response rate among people aged 65+, using this method, was 70%; there were no refusals to answer any of the questions (AB had asked interviewers to be sensitive to respondents if they were hesitant to answer the WHOQOL Death and Dying sub-scale).

The Ethnibus response rate was correctly anticipated at 70% for achieving targets of 400 overall (200 per wave). Box 1 shows the number of addresses targeted and the number of interviews achieved in the 2 survey waves combined.

Box 1. Ethnibus: target interviews by ethnic group re: Census and achieved: combined sample (waves in parenthesis).

Ethnicity	No. Addresses approached	No. required re: Census	Census %	Actual No. Achieved (400):	Response Rate % (both waves identical %)
Indian	200<br>wave 1:100,<br>wave 2:100	152 (38%)	(38%)	152<br>wave 1: 76,<br>wave 2: 76	76%
Pakistani	162<br>wave 1: 80;<br>wave 2: 82	117 (29%)	(29%)	117<br>wave 1: 58,<br>wave 2: 59	72%

Caribbean	159<br>wave 1: 80,<br>wave 2: 79	86 (25%) (21.5%)	86<br>wave 1: 43,<br>wave 2: 43	54%
Chinese	82<br>wave 1: 40,<br>wave 2: 42	45 (8%) (11%)	45<br>wave 1: 22,<br>wave 2: 23	55%

Response for all ethnic groups: 152+117+86+45/200+162+159+82=400/603=66%.

The age distributions of the Ethnibus sample was younger than that of the Omnibus sample (see later), reflecting the fact that people in ethnic minority groups in Britain are younger than the white British population (e.g. estimates from the 2001 census show that while around 17% of the white British population were aged 65+, only about 6% of non-white ethnic minority groups were. Census figures also show that Pakistani and Bangladeshi populations have a younger profile than the Indian population, and Black Caribbean people have the oldest profile. Sample weights were provided by Ethnibus (weighting so that the sample matched the socio-demographic characteristics of ethnic populations aged 65+, using population estimates derived from the last Census). The weightings made no difference at to the sample estimates or further analyses

ONS Omnibus sample

The same questionnaire used in the Ethnibus survey, with the OPQOL, as well as the WHOQOL-OLD and CASP_19, along with standard ONS socio-demographic items, and

our survey questions on active ageing, health and social circumstances, was administered to respondents aged 65+ to two waves of Office for National Statistics (ONS) Omnibus surveys in Britain. The survey conducts face to face interviews with approximately 1200 adults aged 16 or over, living in private households in Britain, each month. The sampling frame used for Omnibus Surveys was the British Postcode Address File (PAF) of 'small users' (all private household addresses). A new sample of 67 postal sectors is selected for each month and stratified by region, the proportion of households where the household reference person in which the head of household is in the National Statistics Socio-economic Classification (NS-=SEC) (Goldthorpe 1980; 1997) categories 1-3 (i.e. employers in large organisations, higher managerial occupations, higher professional employees/self-employed), and the proportion of people who are aged over 65. The postal sectors were selected with probability proportional to size. If an address contains more than one household, the interviewer uses a standard ONS procedure to randomly select where to interview – this may be at one, two or three households depending on the exact circumstances. Within households with more than one adult member, just one person aged 16 or over is selected with the use of a Kish Grid. The interviewers endeavour to interview that person - proxy interviews are not taken.

ONS interviewers identified 589 respondents aged 65+ during the Omnibus interviews in December 2007 and January 2008, and administered our module to all of them (100% agreement to participate). They were representative of the population of Britain in relation to age and sex, when compared with population estimates from the last census.

As with all National Statistics surveys, a quality check on field work was carried out through recall interviews with a proportion of respondents to make sure that the interviews actually took place with those respondents and that responses to questions were consistent. The Office of National Statistics does not collect information about the non-responders. They inform users that the responders are broadly representative of mid-year population estimates, but provide no other information.

The combined response rate for the two Omnibus survey waves was 62% (2256 achieved interviews out of 3660 eligible base; 589 of these respondents were aged 65+) (this represented 61% (n 1130 achieved interviews out of the eligible sample (1864)) in wave 1 (December 2007); 288 of these were aged 65+ - 100% were administered our module; and 63% (n 1126 achieved interviews out of the eligible sample (1796) in wave 2 January 2008; 301 of these were aged 65+ - 100% were administered our module); 1% (23) of households at wave 1 and 1% (14) wave 2 were households unknown to be eligible: combined rate 23+14/3660=1%.Of the eligible households, 30% refused to [participate (553) in wave 1 and also in wave 2 (540); the combined refusal rate was 553+540=1093/3660=30%. There was no contact with 8% (158) in wave 1 and 6% (116) in wave 2; combined no contact rate 158+116=274/3660=7%.

Box 2 shows the response rates for the full Omnibus sample using the base as the number of eligible households. Although the full Omnibus sample comprised 2010 addresses, response rates are calculated using the number of eligible households only. It also shows the numbers of people aged 65+ who were included (100% were administered our

module successfully). The Omnibus sample was representative of the population of Britain in relation to age and sex. Because only one household member is interviewed, people in households containing few adults have a greater chance of selection than those in households with more, a weight is applied to correct for this unequal probability in analyses which use the individual adult as the unit of analysis, and is calculated by dividing the number of adults in the sampled

household by the average number of adults per household. The base is then

adjusted back to the number of respondents who were interviewed. The weightings made no difference to the sample estimates or further analyses.

**Box 2. Response rates for the full Omnibus sample to our module on active aging and QoL (AA-QoL)**			
	**Wave 1 (Dec. 2007)**	**Wave 2 (Jan 2008)**	**Merged sample**
Response rate %	61 %	63 %	62 %
Response rate(count)	1130	1126	2256
Base	1864	1796	3660
	Wave 1 (Dec. 2007)	Wave 2 (Jan 2008)	Merged sample
Response rate to AA-QoL module	288 (100%)	301 (100%)	589 (100%)
**Base: sample of responders aged 65+ to Omnibus Survey**	**288 (representing 25% of 1130 sample of adults of all ages)**	**301 (representing 27% of 1126 sample of adults of all ages)**	**589 (representing 26% of 2256 sample of adults of all ages)**

QoL follow-up study of people aged 65+ at baseline, response rate (2007-8)

The QoL Survey sample was derived from four quarterly Omnibus face-to-face interview surveys of randomly selected private households (via post code files) in Britain, in 1999-2000 carried out by the Office for National Statistics (ONS), using a small-user postcode sampling frame, with geographic and socio-economic stratification. The socio-demographic characteristics of the sample were comparable to those from mid-year population estimates. Omnibus sample members aged 65 and more years were asked whether they would be willing to be re-interviewed about their quality of life, and 999 (77% of those eligible) people aged 65+ were successfully re-interviewed, by ONS interviewers, for the QoL Survey (2000-2001). Of the 1299 eligible respondents, 77% (999) were successfully re-interviewed, 19% refused to participate, and 4% were not contactable during the interview period. Responders and non-responders had similar profiles.

The characteristics of the baseline sample were broadly representative of people aged 65 and over living at home in Britain and have been reported in detail (Bowling 2005). Full details of the baseline and first follow-up samples and methods have been published elsewhere (Bowling et al. 2002; 2003; Bowling & Gabriel 2004; Gabriel & Bowling 2004;Bowling 2005; Bowling et al. 2005).

Of the 999 baseline survey responders in 1999-2000, 770 surviving sample members consented to further follow-up, and were contacted 18 months later with a postal questionnaire about QoL, changes in health and social circumstances. The addresses of non-survivors were removed after checks at NHS Central Registry. The response rate, with two reminders was 69% (Bowling et al. 2005). Survivors were sent a further, brief questionnaire in 2006 (n=564). Of the 338 (60%) who replied, 299 (89%) consented to help us further. We asked these respondents if they would volunteer to help us pre-test the QoL questionnaire; 179 (60%) completed and returned it. No reminders were sent in order to minimise respondent burden.

Survivors aged 65+ at baseline were mailed a further postal questionnaire in 2007-8 (n-=553), containing the OPQOL, plus questions about active ageing, their health, psych-social and economic circumstances. Of these 553 mailings, relatives replied and informed us that a further five sample members had died, and the Royal Mail returned a further 52 envelopes as ‘person not at/unknown at that address’ (suggesting further deaths, as well as moves to residential/care homes or to be with/nearer relatives). A total of 287 completed questionnaires were returned by respondents. The raw response rate, then, was 287 out of 553 mailed: 52%. The response was 52% if deaths were removed from the denominator (302/553 minus 5 deaths=287/548). The valid response rate of 287 questionnaires returned out of 496 valid addresses (removing both 5 deaths and 52 untraced respondents from the denominator =base =496) was 58%. Sample attrition is inevitable in longitudinal surveys, especially in older sample members, where the most vulnerable and ill members of the sample will have died or dropped out, leaving the healthiest sample members. The sample (287) was initially weighted by ONS to correct

for the unequal probability of small households (in which people aged 65+ usually live) being included in the sample and this increased the effective sample size to n= 302.

The follow-up sample was inevitably a sample of survivors, and inevitably biased. However, they form a pragmatic sample, sufficient for the initial testing of the questionnaire which was developed from the baseline responses.
