# Supplementary material for: The Psychometric Properties of the Older People's Quality of Life Questionnaire, Compared with the CASP-19 and the WHOQOL-OLD
Source: Curr Gerontol Geriatr Res. 2010 Feb 1;2009:298950. doi: 10.1155/2009/298950 (PMC2819744; doi:10.1155/2009/298950)
Supplement: Supplementary file 2 [file 298950.f2.pdf]

**Supplementary file Table 1. Older people's Quality of Life Questionnaire (OPQOL) items+**			
	**Ethnibus 2008**<br><br>**(35 items, range 35-175)**	**ONS Omnibus 2008**<br><br>**(35 items, range 35-175)**	**QoL Survey follow-up 2007/8**<br><br>**(32 items, range 32-160)**
**OPQOL items:**	**% (n)**	**% (n)**	**% (n)**
**1. Life overall:**			
1. + I enjoy my life overall			
Strongly agree	18 (73)	38 (224)	21 (59) ****
Agree	34 (137)	53 (310)	61 (171)
Neither agree nor disagree	37 (148)	7 (42)	14 (40)
Disagree	9 (35)	2 (11)	4 (12)
Strongly disagree	2 (7)	---	
2. + I am happy much of the time			
Strongly agree	10 (41)	34 (197)	18 (50)****
Agree	32 (126)	59 (344)	66 (184)
Neither agree nor disagree	49 (196)	5 (32)	12 (32)
Disagree	8 (33)	2 (13)	4 (12)
Strongly disagree	1 (4)	---	---
3. + I look forward to things			
Strongly agree	15 (60)	36 (209)	21 (57) ****
Agree	42 (166)	53 (312)	58 (163)
Neither agree nor disagree	38 (152)	8 (47)	17 (48)

Disagree	5 (21)	3 (17)	4 (11)
Strongly disagree	--- (1)	--- (2)	---
4. - Life gets me down			
Strongly agree	14 (57)	1 (8)	2 (4) ****
Agree	47 (187)	12 (72)	7 (20)
Neither agree nor disagree	23 (92)	17 (100)	25 (69)
Disagree	12 (48)	48 (279)	44 (118)
Strongly disagree	4 (16)	22 (127)	22 (61)
**2. Health and functioning:**			
5. + I have a lot of physical energy			
Strongly agree	13 (53)	12 (71)	6 (17) ****
Agree	41 (165)	34 (202)	28 (76)
Neither agree nor disagree	30 (121)	19 (113)	36 (96)
Disagree	13 (51)	29 (164)	27 (74)
Strongly disagree	3 (10)	6 (36)	3 (9)
6. - Pain affects my well-being±			
Strongly agree	14 (56)	8 (50)	8 (21) ****
Agree	40 (161)	29 (169)	28 (76)
Neither agree nor disagree	32 (128)	15 (86)	21 (56)
Disagree	10 (39)	35 (205)	33 (89)
Strongly disagree	4 (16)	13 (77)	10 (28)
7. - My health restricts me looking after myself or my home			

Strongly agree	15 (61)	4 (26)	5 (13) ****
Agree	33 (133)	15 (88)	12 (34)
Neither agree nor disagree	37 (148)	7 (39)	14 (39)
Disagree	11 (44)	47 (276)	43 (119)
Strongly disagree	4 (14)	27 (158)	26 (71)
8. + I am healthy enough to get out and about			
Strongly agree	8 (30)	39 (227)	29 (81) ****
Agree	32 (130)	47 (276)	51 (144)
Neither agree nor disagree	48 (192)	5 (31)	9 (24)
Disagree	11 (44)	5 (30)	8 (21)
Strongly disagree	1 (4)	4 (23)	3 (10)
**3a. Social relationships:**			
9. + My family, friends or neighbours would help me if needed			
Strongly agree	6 (26)	49 (286)	44 (125) ****
Agree	31 (123)	45 (263)	49 (138)
Neither agree nor disagree	55 (219)	4 (23)	6 (18)
Disagree	8 (31)	2 (12)	--- (1)
Strongly disagree	--- (1)	--- (2)	--- (1)
10. - I would like more companionship or contact with other people			
Strongly agree	3 (13)	4 (21)	4 (10) ****
Agree	33 (134)	16 (95)	19 (53)

Neither agree nor disagree	42 (167)	27 (157)	42 (117)
Disagree	20 (79)	46 (272)	29 (79)
Strongly disagree	2 (7)	7 (41)	6 (17)
11. + I have someone who gives me love and affection			
Strongly agree	10 (38)	50 (297)	45 (125) ****
Agree	45 (178)	38 (222)	35 (96)
Neither agree nor disagree	--- ---	5 (28)	13 (36)
Disagree	44 (177)	5 (29)	6 (17)
Strongly disagree	2 (7)	2 (10)	1 (4)
12/a - I'd like more people to enjoy life with			
Strongly agree	3 (14)	4 (25)	5 (13) ****
Agree	32 (130)	25 (149)	21 (58)
Neither agree nor disagree	41 (164)	27 (157)	42 (116)
Disagree	22 (86)	39 (229)	27 (76)
Strongly disagree	2 (6)	5 (27)	5 (14)
12/b. Aia.± + I have my children around which is important			
Strongly agree	10 (38)	37 (217)	*n/a*
Agree	34 (138)	31 (182)	
Neither agree nor disagree	39 (156)	11 (63)	
Disagree	15 (61)	11 (64)	
Strongly disagree *[includes no children]*	2 (7)	9 (54)	

**4. Independence, control over life, freedom:**			
13. + I am healthy enough to have my independence *(independence- health)*			
Strongly agree	7 (26)	39 (231)	32 (93) ****
Agree	23 (93)	48 (282)	50 (142)
Neither agree nor disagree	41 (163)	5 (25)	9 (26)
Disagree	23 (93)	6 (37)	7 (19)
Strongly disagree	6 (25)	2 (12)	2 (6)
14. + I can please myself what I do *(freedom, autonomy)*			
Strongly agree	10 (39)	42 (246)	30 (84) ****
Agree	45 (180)	48 (281)	53 (151)
Neither agree nor disagree	15 (62)	6 (37)	11 (31)
Disagree	24 (94)	3 (15)	5 (14)
Strongly disagree	6 (25)	1 (8)	1 (4)
15. - The cost of things compared to my pension/income restricts my life *(independence-financial)*			
Strongly agree	12 (48)	11 (64)	8 (23) ****
Agree	15 (60)	28 (162)	23 (65)
Neither agree nor disagree	24 (96)	20 (119)	31 (86)
Disagree	42 (169)	36 (213)	32 (91)
Strongly disagree	7 (27)	5 (29)	6 (18)

16. + I have a lot of control over the important things in my life *(control)*			
Strongly agree	13 (53)	*37 (219)*	26 (74) ****
Agree	41 (163)	*53 (313)*	56 (161)
Neither agree nor disagree	29 (115)	*6 (32)*	14 (40)
Disagree	17 (69)	*4 (22)*	3 (8)
Strongly disagree	--- ---	--- *(1)*	1 (3)
32. - I have responsibilities to others that restrict my social or leisure activities			
Strongly agree	5 (18)	3 (15)	4 (12) ****
Agree	15 (59)	10 (60)	12 (33)
Neither agree nor disagree	20 (82)	7 (40)	16 (44)
Disagree	38 (152)	62 (365)	50 (135)
Strongly disagree	22 (89)	18 (107)	17 (47)
**5. Home and neighbourhood:**			
17. + I feel safe where I live			
Strongly agree	14 (190)	46 (269)	35 (99) ****
Agree	40 (161)	47 (275)	53 (151)
Neither agree nor disagree	9 (36)	4 (24)	9 (26)
Disagree	3 (12)	2 (13)	3 (7)
Strongly disagree	--- (1)	1 (6)	--- (1)
18. + The local shops, services and facilities are good overall			

Strongly agree	32 (127)	22 (132)	23 (64) ****
Agree	54 (216)	50 (293)	59 (168)
Neither agree nor disagree	13 (53)	11 (60)	9 (26)
Disagree	1 (4)	14 (83)	7 (20)
Strongly disagree	--- ---	3 (18)	2 (5)
19. + I get pleasure from my home			
Strongly agree	5 (18)	45 (263)	44 (124) ****
Agree	33 (133)	51 (299)	50 (140)
Neither agree nor disagree	49 (197)	3 (17)	5 (15)
Disagree	12 (48)	1 (8)	1 (4)
Strongly disagree	1 (4)	---	
20. + I find my neighbourhood friendly			
Strongly agree	4 (15)	36 (209)	28 (80) ****
Agree	21 (86)	53 (314)	54 (152)
Neither agree nor disagree	58 (232)	7 (44)	15 (41)
Disagree	16 (62)	3 (16)	3 (8)
Strongly disagree	1 (5)	1 (3)	-- (1)
**6. Psychological and emotional well-being:**			
21. + I take life as it comes and make the best of things			
Strongly agree	14 (57)	43 (256)	35 (98) ****
Agree	45 (179)	51 (302)	58 (164)
Neither agree nor disagree	35 (141)	4 (22)	7 (19)

Disagree	6 (23)	1 (6)	---
Strongly disagree	--- ---	--- (1)	---
22. + I feel lucky compared to most people			
Strongly agree	6 (22)	42 (243)	33 (92) ****
Agree	23 (90)	49 (289)	54 (151)
Neither agree nor disagree	42 (169)	7 (43)	12 (35)
Disagree	24 (98)	2 (11)	1 (3)
Strongly disagree	5 (21)	--- (2)	---
23. + I tend to look on the bright side			
Strongly agree	10 (38)	35 (208)	27 (76) ****
Agree	33 (133)	55 (321)	56 (158)
Neither agree nor disagree	37 (147)	9 (50)	14 (40)
Disagree	20 (82)	1 (8)	2 (6)
Strongly disagree	--- ---	---	---
24. + If my health limits social/leisure activities, then I will compensate and find something else I can do			
Strongly agree	24 (96)	19 (114)	21 (59) ****
Agree	28 (112)	62 (365)	55 (154)
Neither agree nor disagree	30 (121)	14 (83)	22 (62)
Disagree	17 (67)	4 (20)	2 (6)
Strongly disagree	1 (4)	1 (4)	---
**7. Financial circumstances:**			

25. + I have enough money to pay for household bills			
Strongly agree	17 (67)	25 (148)	29 (81) ****
Agree	17 (69)	66 (388)	59 (168)
Neither agree nor disagree	37 (149)	5 (28)	9 (24)
Disagree	25 (99)	3 (18)	3 (10)
Strongly disagree	4 (16)	1 (5)	
26. + I have enough money to pay for household repairs or help needed in the house ∅			
Strongly agree	12 (48)	19 (107)	21 (57) ****
Agree	11 (44)	51 (296)	45 (125)
Neither agree nor disagree	40 (161)	19 (111)	20 (57)
Disagree	31 (124)	9 (53)	13 (37)
Strongly disagree	6 (23)	2 (10)	1 (4)
27. + I can afford to buy what I want to			
Strongly agree	9 (37)	10 (58)	17 (41) ****
Agree	9 (37)	47 (275)	41 (114)
Neither agree nor disagree	38 (153)	19 (110)	23 (64)
Disagree	32 (128)	21 (122)	17 (49)
Strongly disagree	12 (45)	3 (20)	2 (6)
28. - I cannot afford to do things I would enjoy			
Strongly agree	8 (31)	5 (30)	3 (8) ****

Agree	9 (36)	20 (116)	19 (54)
Neither agree nor disagree	28 (112)	20 (117)	28 (77)
Disagree	34 (134)	48 (283)	38 (106)
Strongly disagree	22 (87)	7 (39)	12 (35)
**3b. Leisure and social activities:**			
29. + I have social or leisure activities/hobbies that I enjoy doing			
Strongly agree	8 (30)	28 (165)	20 (57) ****
Agree	36 (144)	51 (300)	54 (149)
Neither agree nor disagree	30 (122)	10 (57)	18 (49)
Disagree	17 (68)	9 (51)	7 (20)
Strongly disagree	9 (36)	2 (13)	1 (4)
30. - I try to stay involved with things			
Strongly agree	8 (32)	26 (154)	18 (51) ****
Agree	30 (120)	57 (335)	57 (157)
Neither agree nor disagree	40 (159)	9 (55)	21 (57)
Disagree	17 (68)	7 (39)	3 (9)
Strongly disagree	5 (21)	1 (3)	1 (4)
31. + I do paid or unpaid work or activities that give me a role in life			
Strongly agree	8 (30)	13 (79)	9 (23) ****
Agree	18 (71)	22 (128)	18 (47)
Neither agree nor disagree	32 (128)	9 (52)	16 (42)

Disagree	30 (121)	41 (239)	42 (113)
Strongly disagree	12 (50)	15 (85)	16 (44)
**8. Religion/culture:**			
Aic.± + Religion, belief or philosophy is important to my QoL			
Strongly agree	11 (45)	18 (106)**	n/a
Agree	47 (187)	37 (216)	
Neither agree nor disagree	23 (92)	21 (125)	
Disagree	16 (65)	18 (105)	
Strongly disagree	3 (11)	6 (35)	
Aid.± + Cultural /religious events/festivals are important to my QoL			
Strongly agree	13 (51)	12 (72)ns	n/a
Agree	51 (205)	29 (169)	
Neither agree nor disagree	23 (93)	23 (135)	
Disagree	11 (42)	30 (174)	
Strongly disagree	2 (9)	6 (37)	
**No. of respondents [item non-response]**	**400** ***[item non-response 0%]***	**585-587** ***[Ø577 for financial item # 26; other item non-response: <1%]***	**269-286** [*item* ***non-response 5-10% (+1 item only=11%]***

+ Please indicate the extent to which you agree or disagree with each of the

following statements

OPQOL: reverse coding of positively worded items (5-1) so higher scores represented higher QoL

ns not statistically significant at least at $p<0.05$ level

***** p<0.01***

******* p<0.0001***

± *Additional items inserted in Ethnibus and ONS Omnibus Surveys*

recommended by Ethnibus focus group members and refined with Ethnibus staff

(QoL follow-up questionnaires had already been mailed out and did not

include these additional items)

± *Ethnibus feedback from interviewers: item 6 QOL is based on physical, psychological and emotional pain due to family and domestic issues. This is a cultural interpretation of the word "pain".*
