# Supplementary material for: The Psychometric Properties of the Older People's Quality of Life Questionnaire, Compared with the CASP-19 and the WHOQOL-OLD
Source: Curr Gerontol Geriatr Res. 2010 Feb 1;2009:298950. doi: 10.1155/2009/298950 (PMC2819744; doi:10.1155/2009/298950)
Supplement: Supplementary file 3 [file 298950.f3.pdf]

**Supplementary file Table 2.** **Control, Autonomy, Pleasure, Self-realisation (CASP-19) items**		
	**Ethnibus 2008**	**ONS Omnibus 2008**
**CASP-19 items:**	**% (n)**	**% (n) ***
**Control:**		
1. - My age prevents me from doing the things I would like to		
Very often	4 (16)	15 (85)****
Sometimes	26 (105)	38 (224)
Not very often	55 (219)	25 (150)
Never	15 (60)	22 (127)
2. - I feel that what happens to me is out of my control		
Very often	6 (23)	9 (50)****
Sometimes	19 (76)	31 (182)
Not very often	51 (206)	33 (194)
Never	24 (95)	27 (158)
3. + I feel free to plan for the future		
Very often	16 (65)	43 (253)****
Sometimes	28 (111)	37 (217)
Not very often	35 (138)	16 (89)
Never	21 (86)	4 (25)
4. - I feel left out of things		

Very often	3 (11)	3 (19)ns
Sometimes	15 (61)	21 (121)
Not very often	47 (189)	27 (158)
Never	35 (139)	49 (287)
**Autonomy:**		
5. + I can do the things that I want to do		
Very often	11 (42)	54 (320)****
Sometimes	49 (198)	36 (210)
Not very often	35 (141)	8 (46)
Never	5 (19)	2 (11)
6. - Family responsibilities prevent me from doing what I want to do		
Very often	--- (2)	1 (9)*
Sometimes	8 (32)	12 (69)
Not very often	52 (207)	29 (168)
Never	40 (159)	58 (340)
7. + I feel that I can please myself what I can do		
Very often	24 (95)	66 (388)****
Sometimes	36 (145)	26 (156)
Not very often	33 (131)	5 (27)
Never	7 (29)	3 (15)
8. - My health stops me from doing the		

things I want to do		
Very often	13 (51)	14 (81)ns
Sometimes	23 (93)	33 (193)
Not very often	39 (154)	22 (132)
Never	25 (102)	31 (180)
9. - Shortage of money stops me from doing the things I want to do		
Very often	10 (39)	10 (56)ns
Sometimes	19 (76)	28 (166)
Not very often	39 (155)	31 (180)
Never	33 (130)	31 (184)
**Pleasure**		
10. + I look forward to each day		
Very often	22 (88)	75 (439)****
Sometimes	49 (197)	21 (121)
Not very often	25 (100)	4 (22)
Never	4 (15)	--- (4)
11. + I feel that my life has meaning		
Very often	13 (52)	66 (390)****
Sometimes	57 (228)	27 (156)
Not very often	26 (103)	5 (30)
Never	4 (17)	2 (9)
12. + I enjoy the things that I do		

Very often	26 (106)	80 (470)****
Sometimes	50 (198)	19 (110)
Not very often	23 (93)	1 (6)
Never	1 (3)	--- (1)
13. + I enjoy being in the company of others		
Very often	28 (112)	71 (418)****
Sometimes	58 (233)	26 (152)
Not very often	13 (52)	3 (16)
Never	1 (3)	--- (1)
14. + On balance, I look back on life with a sense of happiness		
Very often	31 (126)	76 (444)****
Sometimes	44 (177)	21 (123)
Not very often	21 (82)	3 (17)
Never	4 (15)	--- (2)
**Self-realisation:**		
15. + I feel full of energy these days		
Very often	3 (12)	19 (112)****
Sometimes	41 (162)	46 (271)
Not very often	33 (132)	26 (150)
Never	23 (94)	9 (54)
16. + I choose to do things that I have never done before		

Very often	10 (40)	7 (41)ns
Sometimes	23 (94)	34 (202)
Not very often	44 (176)	37 (216)
Never	23 (90)	22 (128)
17. + I feel satisfied with the way my life has turned out		
Very often	15 (59)	56 (326)****
Sometimes	45 (180)	36 (214)
Not very often	34 (137)	6 (34)
Never	6 (24)	2 (12)
18. + I feel that life is full of opportunities		
Very often	4 (17)	34 (195)****
Sometimes	22 (90)	38 (223)
Not very often	54 (215)	23 (136)
Never	20 (78)	5 (31)
19. + I feel that the future looks good for me		
Very often	6 (24)	32 (186)****
Sometimes	39 (158)	46 (271)
Not very often	45 (180)	17 (97)
Never	10 (38)	5 (30)
**No. of respondents [item non-response]**	**400** ***[item non-response 0%]***	**583-587** ***[item non-response 1%]***

- Note CASP-19 reverse coding of positively worded items (3-0), so higher scores representing higher QoL

ns not statistically significant at least at $p<0.05$ level

** $p<0.05$*

***** $P<0.0001$*
