# Supplementary material for: The Psychometric Properties of the Older People's Quality of Life Questionnaire, Compared with the CASP-19 and the WHOQOL-OLD
Source: Curr Gerontol Geriatr Res. 2010 Feb 1;2009:298950. doi: 10.1155/2009/298950 (PMC2819744; doi:10.1155/2009/298950)
Supplement: Supplementary file 4 [file 298950.f4.pdf]

**Supplementary file Table 3.** WHOQOL-OLD items		
	**Ethnibus 2008**	**ONS Omnibus 2008**
**WHOQOL-OLD items:**	**% (n)**	**% (n)**
**Sensory abilities:**		
1. + To what extent do impairments to your senses (e.g. hearing, vision, taste, smell, touch) affect your daily life?		
Not at all	12 (46)	51 (297)****
A little	55 (218)	31 (183)
A moderate amount	24 (97)	11 (66)
Very much	8 (32)	6 (35)
An extreme amount	1 (7)	1 (6)
2. + To what extent does loss of, for example, hearing, vision, taste, smell or touch affect your ability to participate in activities?		
Not at all	12 (49)	61 (360)****
A little	58 (231)	20 (119)
A moderate amount	26 (105)	10 (58)
Very much	3 (10)	8 (45)

An extreme amount	---	1 (5)
10. + To what extent do problems with your sensory functioning (e.g. hearing, vision, taste, smell, touch) affect your ability to interact with others?		
Not at all	11 (46)	65 (383)****
A little	59 (234)	22 (127)
Moderately	25 (99)	8 (47)
Mostly	5 (19)	4 (21)
Completely	---	1 (7)
20. - How would you rate your sensory functioning (e.g. hearing, vision, taste, smell, touch)?		
Very poor	2 (7)	4 (21) ns
Poor	5 (19)	7 (44)
Neither poor nor good	22 (90)	18 (103)
Good	46 (184)	47 (274)
Very good	25 (100)	24 (143)
**Autonomy:**		
3. - How much freedom do you have to make your own decisions?		
Not at all *(sic)*	15 (61)	2 (13)****
A little	12 (49)	5 (29)

A moderate amount	19 (75)	13 (77)
Very much	28 (110)	52 (302)
An extreme amount	26 (105)	28 (166)
4. - To what extent do you feel in control of your future?		
Not at all	1 (5)	6 (33)**
A little	8 (31)	10 (58)
A moderate amount	24 (97)	21 (124)
Very much	43 (172)	51 (292)
An extreme amount	24 (95)	12 (70)
5. - How much do you feel that the people around you are respectful of your freedom?		
Not at all	3 (13)	2 (14) ns
A little	7 (28)	5 (29)
A moderate amount	32 (127)	18 (103)
Very much	39 (155)	59 (344)
An extreme amount	19 (77)	15 (87)
11. - To what extent are you able to do the things you'd like to do?		
Not at all	3 (10)	6 (38)**
A little	14 (54)	14 (84)
Moderately	37 (150)	19 (108)

Mostly	31 (125)	42 (241)
Completely	15 (61)	19 (114)
**Past, present and future abilities:**		
12. - To what extent are you satisfied with your opportunities to continue achieving in life?		
Not at all	2 (8)	8 (46)****
A little	11 (43)	12 (68)
Moderately	31 (126)	25 (145)
Mostly	37 (149)	38 (221)
Completely	19 (74)	17 (98)
13. - How much do you feel that you have received the recognition you deserve in life?		
Not at all	4 (16)	4 (23) ns
A little	16 (64)	14 (75)
Moderately	35 (140)	29 (164)
Mostly	32 (127)	37 (213)
Completely	13 (53)	17 (99)
15. - How satisfied with what you have achieved in life?		
Very dissatisfied	14 (56)	6 (33)**
Dissatisfied	7 (27)	4 (21)

Neither satisfied nor dissatisfied	17 (70)	11 (62)
Satisfied	30 (118)	53 (313)
Very satisfied	32 (129)	26 (155)

19. - How happy are you with the things you are able to look forward to?		
Very unhappy	2 (7)	4 (23)*
Unhappy	6 (25)	3 (19)
Neither happy nor unhappy	22 (88)	16 (91)
Happy	38 (152)	50 (296)
Very happy	32 (128)	27 (156)
**Social participation**		
14. - To what extent do you feel that you have enough to do each day?		
Not at all	2 (7)	1 (7) ns
A little	11 (44)	8 (47)
Moderately	39 (157)	16 (93)
Mostly	29 (115)	43 (252)
Completely	19 (77)	32 (184)
16. - How satisfied are you with the way you use your time?		
Very dissatisfied	7 (26)	3 (19)**
Dissatisfied	16 (63)	5 (32)

Neither satisfied nor dissatisfied	26 (104)	15 (85)
Satisfied	28 (114)	56 (328)
Very satisfied	23 (93)	21 (121)
**17. - How satisfied are you with your level of activity?**		
Very dissatisfied	4 (15)	4 (23) ns
Dissatisfied	7 (29)	14 (83)
Neither satisfied nor dissatisfied	29 (118)	13 (78)
Satisfied	38 (150)	51 (298)
Very satisfied	22 (88)	18 (103)
18. - How satisfied are you with your opportunity to participate in community activities		
Very dissatisfied	2 (7)	4 (23) *
Dissatisfied	4 (14)	9 (50)
Neither satisfied nor dissatisfied	32 (130)	30 (173)
Satisfied	39 (157)	43 (254)
Very satisfied	23 (92)	14 (84)
**Death and Dying:**		
6. + How concerned are you about the way in which you will die?		
Not at all	11 (43)	47 (268)**
A little	21 (83)	27 (156)

A moderate amount	33 (134)	15 (87)
Very much	22 (89)	9 (51)
An extreme amount	13 (51)	2 (12)
7. + How much are you afraid of not being able to control your death?+		
Not at all	7 (26)	45 (256)****
Slightly	23 (92)	22 (127)
Moderately	27 (109)	15 (86)
Very	25 (101)	13 (78)
Extremely	18 (72)	4 (23)
8. + How scared are you of dying+		
Not at all	15 (60)	59 (341)****
Slightly	23 (91)	22 (124)
Moderately	29 (117)	11 (66)
Very	16 (65)	5 (30)
Extremely	17 (67)	3 (15)
9. + How much do you fear being in pain before you die?		
Not at all	5 (21)	21 (120) ns
A little	11 (45)	27 (158)
A moderate amount	32 (126)	18 (100)
Very much	37 (148)	26 (146)
An extreme amount	15 (60)	8 (47)

**Intimacy:**		
21. - To what extent do you feel a sense of companionship in your life?		
Not at all	4 (14)	5 (29) ns
A little	8 (31)	12 (68)
A moderate amount	26 (104)	18 (103)
Very much	44 (178)	47 (277)
An extreme amount	18 (73)	18 (104)
22. - To what extent do you experience love in your life?		
Not at all	2 (7)	5 (31)**
A little	11 (43)	11 (62)
A moderate amount	29 (117)	13 (78)
Very much	37 (147)	47 (274)
An extreme amount	21 (86)	24 (139)
23. - To what extent do you have opportunities to love?		
Not at all	5 (19)	8 (46)**
A little	17 (69)	10 (57)
Moderately	30 (119)	19 (110)
Mostly	36 (145)	35 (204)
Completely	12 (48)	28 (165)
24. - To what extent do you have		

opportunities to be loved?		
Not at all	3 (12)	8 (45)***
A little	16 (64)	9 (55)
Moderately	31 (123)	17 (102)
Mostly	42 (170)	38 (221)
Completely	8 (31)	26 (155)
**No. of respondents [item non-response]**	**400 *[item non-response 0%]***	**570-587 *[item non-response 1-3%]***

+ this slight WHOQOL alteration in response categories for these two items was overlooked in error: Not at all, Slightly. Moderately, Very, Extremely

-+Note + WHOQOL-OLD *reverse coding of positively worded items (5-1), with higher scores representing higher QoL*

ns not statistically significant at least at p<0.05 level

** p<0.05*

*** p<0.01*

**** p<0.001*

***** P<0.0001*
