# Supplementary material for: The Psychometric Properties of the Older People's Quality of Life Questionnaire, Compared with the CASP-19 and the WHOQOL-OLD
Source: Curr Gerontol Geriatr Res. 2010 Feb 1;2009:298950. doi: 10.1155/2009/298950 (PMC2819744; doi:10.1155/2009/298950)
Supplement: Supplementary file 5 [file 298950.f5.pdf]

Supplementary file Table 4. OPQOL subscale reliability						
**OPQOL Subscales:** **[Ethnibus, ONS Omnibus 35 items; 5-point scale (1-5) range 35-175; QoL follow-up: 32 items, range 32-160)]**	**Mean for sub-scale score**	**sd for subscale score**	**Subscale score skewness**	**Subscale score kurtosis**	**Inter Subscale Correlation range**	**Corrected Subscale-Total correlation**
**Life overall (4 items; scale range 4-20)**						
Ethnibus	13.108	2.074	0.474	0.552	-0.011 – 0.303	0.314
ONS Omnibus	16.478	2.399	-0.595	0.591	0.086 – 0.559	0.692
QoL follow up	15.685	2.557	-0.536	0.731	0.268 – 0.798	0.761
**Health and functioning (4 items; scale range 4-20)**						
Ethnibus	11.883	2.047	0.152	0.729	0.057 – 0.147	0.188
ONS Omnibus	14.218	3.450	-0.513	-0.201	-0.024 – 0.521	0.553
QoL follow up	14.000	3.235	-0.386	-0.084	0.219 – 0.713	0.648
**Social relationships and participation (8 items; scale range 8-40; NB 7 items for QoL follow-up sample; range 7-35)**						
Ethnibus	25.123	3.438	0.208	0.315	0.047 – 0.560	0.461
ONS Omnibus	29.723	4.285	-0.454	0.571	0.214 – 0.559	0.617
QoL follow up	25.215	3.882	-0.081	0.326	0.363 – 0.791	0.729
**Control, independence, freedom (5 items; range 5-25)**						
Ethnibus	12.958	2.540	-0.250	-0.548	-0.011 – 0.438	0.322
ONS Omnibus	19.467	2.675	-0.419	0.731	-0.006 – 0.521	0.625
QoL follow up	18.624	2.896	-0.331	0.317	0.181 – 0.714	0.657
**Area: Home and**						

**neighbourhood (4 items; range 4-20)**						
Ethnibus	14.875	1.918	0.286	-0.359	0.106 – 0.424	0.427
ONS Omnibus	16.894	2.155	-0.456	0.491	0.070 – 0.458	0.493
QoL follow up	16.632	2.204	-0.579	1.171	0.181 – 0.524	0.463
**Psychological well-being and outlook (4 items; range 4-20)**						
Ethnibus	13.550	2.547	-0.137	-0.125	0.110 – 0.560	0.586
ONS Omnibus	16.889	1.968	-0.192	-0.291	0.180 – 0.553	0.540
QoL follow up	16.442	2.024	0.045	-0.522	0.255 – 0.663	0.463
**Financial circumstances (4 items; range 4-20)**						
Ethnibus	12.363	2.801	-0.110	-0.332	0.047 – 0.438	0.249
ONS Omnibus	14.597	2.822	-0.729	1.170	0.012 – 0.496	0.390
QoL follow up	14.786	3.096	-0.251	-0.308	0.275 – 0.668	0.600
**Religion/culture (2 items; range 2-10)**						
Ethnibus	7.093	1.674	-0.527	0.055	0.035 – 0.256	0.271
ONS Omnibus	6.542	2.118	-0.179	-0.651	-0.006 – 0.214	0.101
QoL follow up	n/a	n/a	n/a	n/a	n/a	n/a
