# Supplementary material for: The Psychometric Properties of the Older People's Quality of Life Questionnaire, Compared with the CASP-19 and the WHOQOL-OLD
Source: Curr Gerontol Geriatr Res. 2010 Feb 1;2009:298950. doi: 10.1155/2009/298950 (PMC2819744; doi:10.1155/2009/298950)
Supplement: Supplementary file 6 [file 298950.f6.pdf]

**Supplementary file Table 5. CASP-19 subscale reliability**						
**CASP -19 Subscales:** **Casp-19 TOTAL** **[19 items; 5-point (0-3) scale range 0-57)**	**Mean for sub-scale**	**sd for subscale**	**Subscale skewness**	**Subscale kurtosis**	**Inter Subscale Correlation range**	**Corrected Subscale-Total correlation**
**Control (4 items; scale range 0-12)**						
Ethnibus	7.767	1.714	-0.109	-0.005	0.136 – 0.382	0.349
ONS Omnibus	7.881	2.601	-0.334	0.532	0.433 – 0.662	0.684
**Autonomy (5 items; scale range 0-15)**						
Ethnibus	9.437	2.196	-0.342	0.420	0.135 – 0.382	0.316
ONS Omnibus	11.000	2.607	-0.461	0.013	0.407 – 0.662	0.655
**Pleasure: (5 items; scale range 0-15)**						
Ethnibus	19.870	2.162	-0.096	0.686	0.135 – 0.258	0.269
ONS Omnibus	13.502	1.896	-1.411	1.563	0.407 – 0.537	0.547
**Self-realisation: (5 items; scale range 0-15)**						
Ethnibus	6.660	2.220	0.147	0.228	0.136 – 0.258	0.269
ONS Omnibus	9.574	2.842	-0.483	0.024	0.519 – 0.549	0.647
