# Supplementary material for: The Psychometric Properties of the Older People's Quality of Life Questionnaire, Compared with the CASP-19 and the WHOQOL-OLD
Source: Curr Gerontol Geriatr Res. 2010 Feb 1;2009:298950. doi: 10.1155/2009/298950 (PMC2819744; doi:10.1155/2009/298950)
Supplement: Supplementary file 7 [file 298950.f7.pdf]

**Supplementary file Table 6.** **WHOQOL-old subscale reliability**						
**WHOQOL Subscales:** **[24 items; 5-point (1-5) scale range 24-120**	**Mean for sub-scale**	**sd for subscale**	**Subscale skewness**	**Subscale kurtosis**	**Inter Subscale Correlation range**	**Corrected Subscale-Total correlation**
**Sensory ability SAB (4 items; scale range 4-20)**						
Ethnibus	15.067	2.228	-0.459	0.995	-0.011 – 0.141	0.032
ONS Omnibus	16.869	3.164	-1.273	1.177	0.043 – 0.395	0.363
**Autonomy AUT 4 items; scale range)**						
Ethnibus	14.245	2.277	-0.289	0.109	-0.200 – 0.097	0.030
ONS Omnibus	14.906	2.772	-0.530	0.219	0.095 – 0.554	0.533
**Present and future activities PPF (4 items; scale range 4-20)**						
Ethnibus	14.452	2.440	-0.233	-0.232	-0.111 – 0.225	0.229
ONS Omnibus	14.852	2.712	-0.349	-0.220	0.129 – 0.576	0.626
**Social participation SOP SOP (4 items; scale range 4-20)**						
Ethnibus	14.440	2.220	0.032	-0.400	-0.044 – 0.225	0.156
ONS Omnibus	15.084	2.842	-0.608	0.412	0.123 – 0.576	0.551
**Death and Dying DAD**						

**(4 items; scale range 4-20)**<br>Ethnibus	11.270	3.256	0.026	-0.173	-0.200 – 0.016	-0.154
ONS Omnibus	15.428	3.648	-0.751	-0.004	0.034 – 0.129	0.115
**Intimacy INT (4 items; scale range 4-20)**<br>Ethnibus	14.012	2.626	-0.573	0.530	-0.069 – 0.155	0.098
ONS Omnibus	14.779	4.022	-0.786	-0.057	0.034 – 0.387	0.310
