# Supplementary material for: The Psychometric Properties of the Older People's Quality of Life Questionnaire, Compared with the CASP-19 and the WHOQOL-OLD
Source: Curr Gerontol Geriatr Res. 2010 Feb 1;2009:298950. doi: 10.1155/2009/298950 (PMC2819744; doi:10.1155/2009/298950)
Supplement: Supplementary file 8 [file 298950.f8.pdf]

Supplementary file Table 7. OPQOL and CASP-19 subscale and total correlations (Spearman's rho)

	**CASP-19 [19 items]**				
	**CONTROL**	**AUTONOMY**	**PLEASURE**	**SELF REALISATION**	**TOTAL SCORE**
**OPQOL total and sub-scales[35 items]**					
**OPQOL Total**					
Ethnibus	0.193**	0.294**	0.418**	0.344**	0.488**
ONS Omnibus	0.573**	0.600**	0.551**	0.653**	0.739**
**Life overall**					
Ethnibus	-0.047	-0.070	0.088	0.147**	0.031
ONS Omnibus	0.500**	0.477**	0.512**	0.543**	0.621**
**Health and functioning**					
Ethnibus	0.080	0.135**	0.087	0.056	0.128**
ONS Omnibus	0.534**	0.549**	0.316**	0.611**	0.655**
**Social relationships and participation**					
Ethnibus	-0.043	-0.003	0.300**	0.285**	0.194**
ONS Omnibus	0.484**	0.366**	0.453**	0.522**	0.569**
**Control, independence, freedom**					
Ethnibus	0.308**	0.406**	0.378**	0.265**	0.537**
ONS Omnibus	0.430**	0.604**	0.337**	0.393**	0.549**

**Area: Home and neighbourhood**					
Ethnibus	0.125*	0.030	0.334**	0.197**	0.269**
ONS Omnibus	0.275**	0.217**	0.373**	0.347**	0.372**
**Psychological well-being and outlook**					
Ethnibus	0.040	0.142**	0.273**	0.311**	0.279**
ONS Omnibus	0.345**	0.274**	0.416**	0.354**	0.415**
**Financial circumstances**					
Ethnibus	0.289**	0.412**	0.225**	0.078	0.388**
ONS Omnibus	0.300**	0.500**	0.248**	0.267**	0.411**
**Religion/culture**					
Ethnibus	0.045	0.057	0.214**	0.173**	0.193**
ONS Omnibus	0.001	-0.038	0.149**	0.141**	0.064

* $p<0.05$ ** $p<0.01$
