# Supplementary material for: The Psychometric Properties of the Older People's Quality of Life Questionnaire, Compared with the CASP-19 and the WHOQOL-OLD
Source: Curr Gerontol Geriatr Res. 2010 Feb 1;2009:298950. doi: 10.1155/2009/298950 (PMC2819744; doi:10.1155/2009/298950)
Supplement: Supplementary file 9 [file 298950.f9.pdf]

Supplementary file Table 8. OPQOL and WHOQOL-OLD subscale and total correlations (Spearman's rho)

	WHOQOL-OLD [24 items]:						
**OPQOL [35 items]:**	**Sensory abilities SAB**	**Autonomy AUT**	**Past, present and future Activities PPF**	**Social participation SOP**	**Death and dying DAD**	**Intimacy INT**	**TOTAL SCORE**
**OPQOL Total**							
Ethnibus	0.313**	0.009	0.148**	0.134**	0.334**	0.028	0.405**
ONS Omnibus	0.336**	0.532**	0.584**	0.601**	0.144**	0.428**	0.699**
**Life overall**							
Ethnibus	0.174**	0.009	0.125**	0.119*	0.025	-0.040	0.148**
ONS Omnibus	0.263**	0.435**	0.483**	0.468**	0.156**	0.349**	0.559**
**Health and functioning**							
Ethnibus	0.108*	-0.017	-0.096	-0.005	0.121*	-0.028	0.054
ONS Omnibus	0.440**	0.441**	0.403**	0.541**	0.028	0.210**	0.527**
**Social relationships and participation**							
Ethnibus	0.105*	-.118	0.048	0.080	0.358**	-0.001	0.222**
ONS Omnibus	0.279**	0.414**	0.472**	0.530**	0.045	0.503**	0.624**
**Control over life, independence, freedom**							
Ethnibus	0.275**	0.070	0.123*	0.056	0.152**	-0.056	0.235**

ONS Omnibus	0.268**	0.472**	0.372**	0.386**	0.152**	0.142**	0.456**
**Area: Home and neighbourhood**							
Ethnibus	0.186**	0.043	0.187**	0.075	0.252**	0.042	0.298**
ONS Omnibus	0.196**	0.272**	0.365**	0.292**	0.152**	0.318**	0.421**
**Psychological well-being and outlook**							
Ethnibus	0.143**	-0.025	0.099*	0.110*	0.407**	0.038	0.326**
ONS Omnibus	0.186**	0.339**	0.412**	0.377**	0.149**	0.309**	0.462**
**Financial circumstances**							
Ethnibus	0.250**	0.134**	0.184**	0.151**	-0.050	-0.101	0.207**
ONS Omnibus	0.086*	0.265**	0.288**	0.232**	0.124**	0.122**	0.316**
**Religion/culture**							
Ethnibus	0.220**	-0.004	0.038	-0.004	0.214**	0.192**	0.264**
ONS Omnibus	-0.012	0.072	0.162**	0.125**	0.021	0.163**	0.148**

* $p<0.05$ ** $p<0.01$
