# Supplementary material for: The Psychometric Properties of the Older People's Quality of Life Questionnaire, Compared with the CASP-19 and the WHOQOL-OLD
Source: Curr Gerontol Geriatr Res. 2010 Feb 1;2009:298950. doi: 10.1155/2009/298950 (PMC2819744; doi:10.1155/2009/298950)
Supplement: Supplementary file 10 [file 298950.f10.pdf]

Supplementary file Table 9. CASP-19 and WHOQOL-OLD subscale and total correlations (Spearman's rho)

	WHOQOL -OLD:						
**CASP-19**	**Sensory abilities SAB**	**Auton-omy AUT**	**Past, present and future activities PPF**	**Social partici-pation SOP**	**Death and Dying DAD**	**Intim-acy INT**	**TOTAL SCORE**
**CONTROL**							
Ethnibus	0.128*	0.172**	0.232**	0.144**	-0.038	0.209**	0.301**
ONS Omnibus	0.384**	0.522**	0.482**	0.507**	0.171**	0.248**	0.599**
**AUTONOMY**							
Ethnibus	0.199**	0.137**	0.136**	0.184**	-0.067	0.129**	0.239**
ONS Omnibus	0.347**	0.526**	0.432**	0.438**	0.197**	0.127**	0.526**
**PLEASURE**							
Ethnibus	0.197**	-0.011	0.143**	0.020	0.194**	0.020	0.225**
ONS Omnibus	0.213**	0.367**	0.459**	0.368**	0.099*	0.414**	0.543**
**SELF-REALISATION**							
Ethnibus	0.078	-0.044	0.017	0.029	0.318**	0.158**	0.253**
ONS Omnibus	0.314**	0.473**	0.547**	0.528**	0.056	0.348**	0.607**
**TOTAL SCORE**							
Ethnibus	0.243**	0.087	0.193**	0.126*	0.146**	0.199**	0.380**
ONS Omnibus	0.403**	0.590**	0.588**	0.581**	0.152**	0.340**	0.694**

n Ethnibus: 400; ONS: 573;
** p<0.05 ** p<0.01*
